# Magnetic microfiber hyperthermia for synergistic antimicrobial activity against methicillin-resistant *Staphylococcus aureus*

**DOI:** 10.1016/j.mtbio.2025.101862

**Published:** 2025-05-12

**Authors:** Shaquib Rahman Ansari, Dominique Grimm, Reshma V. Ramachandran, Yael del Carmen Suárez-López, Krisztina Juriga-Tóth, Georgios A. Sotiriou, Alexandra Teleki

**Affiliations:** a Department of Pharmacy, Science for Life Laboratory, Uppsala University, 75123, Uppsala, Sweden; b Department of Microbiology, Tumor and Cell Biology, Karolinska Institutet, 17177, Stockholm, Sweden; c Department of Chemistry, Science for Life Laboratory, Stockholm University, 11418, Stockholm, Sweden

**Keywords:** Antimicrobial resistance, Superparamagnetic iron oxide nanoparticles, Flame spray pyrolysis, Electrospinning, Magnetic heating, Bacterial biofilms

## Abstract

Methicillin-resistant *Staphylococcus aureus* (MRSA) poses a significant global healthcare challenge, causing a range of life-threatening infections, including osteomyelitis, septic arthritis, skin and soft tissue infections, and wound infections. These infections are difficult to treat, often requiring aggressive therapeutic strategies at high antibiotic doses that increase the risk of adverse effects and drive the development of antimicrobial resistance. An alternative strategy to enhance antibiotic efficacy involves the use of locally elevated temperatures to increase the bacterial susceptibility to drugs. This can be achieved non-invasively, using magnetic hyperthermia induced by superparamagnetic iron oxide nanoparticles (SPIONs) in an alternating magnetic field (AMF). This study, presents a synergistic platform combining magnetic hyperthermia and antibiotic therapy to combat MRSA infections. Magnetic microfibers were fabricated by electrospinning using poly(methyl methacrylate) and tributyl citrate, incorporating functional Mn_0.25_Fe_2.75_O_4_ nanoparticles. The microfibers were systematically optimized to attain necessary tensile strength and heating efficiency for localized treatment of MRSA. Upon AMF exposure, the SPION-loaded microfiber discs achieved tunable temperatures exceeding 60 °C, controlled by varying the microfiber disc weight. The combination of doxycycline and magnetic hyperthermia exposure for 15 min demonstrated significant synergistic effects against MRSA at temperatures above 50 °C. *In vitro*, the antibiotic efficacy of doxycycline was enhanced by up to 35 % against MRSA, even at sub-inhibitory drug doses. The use of biocompatible materials in magnetic microfibers makes them well suited for localized therapy, particularly for treating wound infections. Additionally, the synergistic combination of magnetic hyperthermia with antibiotic therapy could enable lower drug doses, reducing the antibiotic burden and helping to combat antimicrobial resistance.

## Graphical abstract

**Figure:**
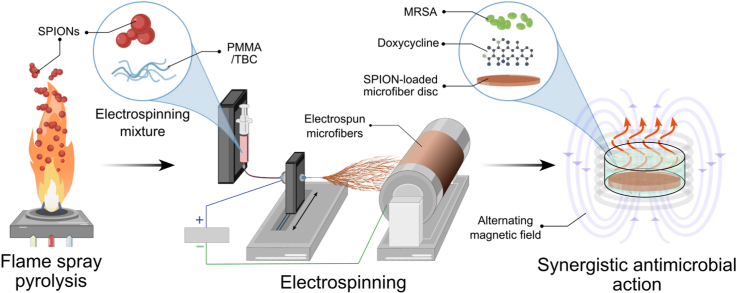

## 1 Introduction

The global prevalence of methicillin-resistant *Staphylococcus aureus* (MRSA) has emerged as a formidable challenge due to its ability to cause life-threatening infections, in both healthcare and community settings [1]. Approximately half a million individuals in the United States alone contract a staphylococcal infection each year resulting in several thousand deaths, with healthcare expenses surpassing $14 billion [2]. MRSA strains, distinguished by their resistance to beta-lactam antibiotics, can cause a spectrum of infections, including those affecting the skin, soft tissues, implantable scaffolds, and severe systemic diseases [2]. Furthermore, the ability of MRSA to form biofilms on surfaces such as surgical implants, makes it more resistant to treatment due to the reduced penetration of antibiotics through the biofilm [3]. Conventional treatments for MRSA infections often rely on antibiotics such as vancomycin and doxycycline, which in high doses can have serious adverse reactions such as kidney damage, thrombophlebitis, and epidermal necrolysis [2]. Furthermore, prolonged use of these drugs can lead to the development of antibiotic-resistance. The growing limitations of the current treatments therefore necessitate the need for improved therapeutic strategies against MRSA.

The treatment of bacterial infections has advanced with the development of innovative therapeutic strategies including ultrasound-based interventions, hyperthermia, photodynamic therapy, bacteriophage therapy, and the use of inorganic nanoparticles [[4], [5], [6], [7], [8]]. Among these, hyperthermia therapy, characterized by the application of elevated temperatures, has demonstrated potent bactericidal effects and the capacity to disrupt adherent biofilms [[9], [10], [11]]. Temperatures above 50 °C can significantly enhance the antimicrobial activity of drugs by weakening bacterial membranes and eliciting an inflammatory response [12]. This approach has been particularly effective against MRSA, where photothermal therapy has shown enhanced antibacterial efficacy in combination with antibiotics [13,14].

Superparamagnetic iron oxide nanoparticles (SPIONs) offer a promising approach to implement hyperthermia therapy against MRSA. Their ability to be magnetized only in the presence of an external magnetic field, and to release heat under an alternating magnetic field (AMF), makes them highly suitable for localized hyperthermia applications. Moreover, SPIONs are approved by the US Food and Drug Administration and the European Medicines Agency, for use as a contrast agent in magnetic resonance imaging, and in magnetic hyperthermia therapy against glioblastoma [15]. SPIONs have also shown bactericidal activity against strains such as *Staphylococcus aureus*, *Acinetobacter baumannii*, *Porphyromonas gingivalis*, and *Proteus mirabilis* [[16], [17], [18], [19], [20], [21]]. The antibacterial effect is likely a result of oxidative stress or thermal damage induced by magnetic hyperthermia. However, infections caused by drug-resistant bacteria like MRSA may require a more targeted approach, combining localized SPION-mediated heating with site-specific antibiotic delivery.

Localized delivery can be achieved through biocompatible microfibers incorporating SPIONs, designed for sites such as skin, internal organs, bone, and implants [22]. Microfibers exhibit unique characteristics such as high surface-area-to-volume ratio, porous structure, ease of handling, tunable fiber properties, and suitability for scalable production [23]. Previous studies have demonstrated the efficacy of drug-loaded microfibers for treating MRSA infection of skin, allowing precise drug release without adverse effects [24,25]. Similarly, ciprofloxacin and silver nanoparticle-loaded microfibers have been utilized for antibacterial treatment of wounds [26]. These microfibers can be modified to respond to external factors like pH, temperature, light, or magnetic fields by incorporating stimuli responsive nanoparticles [[27], [28], [29]]. The incorporation of SPIONs into microfibers, primarily through electrospinning, has been explored in previous studies [[30], [31], [32], [33]]. However, none have focused on optimizing the heating efficiency of magnetic microfibers, leaving a critical gap in understanding their potential for controlled thermal applications. Moreover, while SPION-loaded fibers have been utilized for drug delivery against glioblastoma [30], their application in combating bacterial infections, particularly MRSA, has not been investigated till date.

**Fig. 1:**
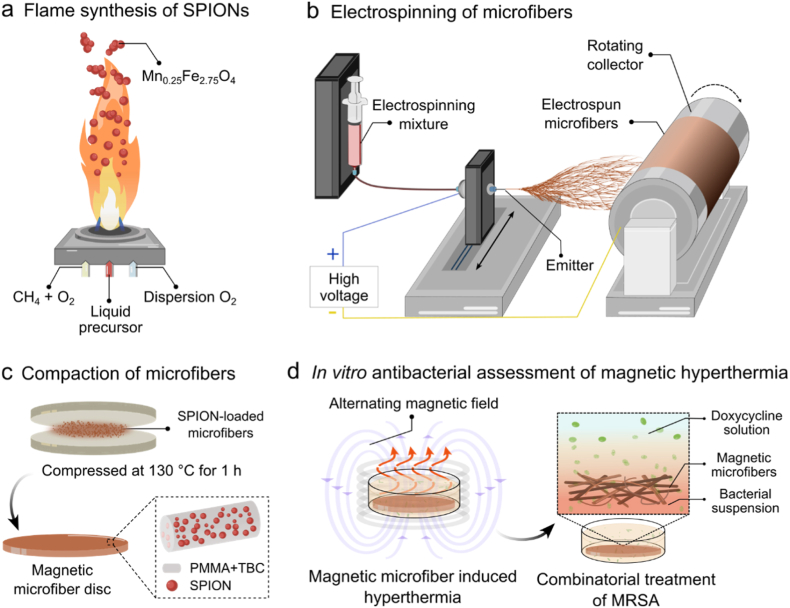
(a) Synthesis of superparamagnetic iron oxide nanoparticles (SPIONs) by flame spray pyrolysis (FSP). (b) Production of magnetic microfibers through electrospinning. (c) Compacted microfiber discs composed of SPIONs, poly(methyl methacrylate) (PMMA), and tributyl citrate (TBC). (d) Antibacterial action of magnetic microfiber and doxycycline against MRSA in an alternating magnetic field.

In this study, we developed SPION-loaded microfibers that deliver magnetic hyperthermia, acting synergistically with doxycycline against MRSA (Fig. 1). Doxycycline was selected as the model drug because it is recommended as a first-line treatment for uncomplicated skin and soft tissue infections [34,35], due to its high bioavailability and favorable safety profile. Its use against vancomycin-resistant MRSA further underscores its relevance in antibiotic resistance [36]. SPIONs were synthesized via flame spray pyrolysis (FSP) (Fig. 1a), a scalable and reproducible nanoparticle manufacturing technique [37]. Electrospinning was used to incorporate the nanoparticles into the microfiber polymer matrix (Fig. 1b), which was composed of poly(methyl methacrylate) (PMMA) and tributyl citrate (Fig. 1c) [33]. The effect of heat and antibiotics on MRSA was systematically investigated. *In vitro* antibacterial activity of magnetic microfiber mediated hyperthermia was evaluated over a range of antibiotic concentrations (Fig. 1d). Finally, multifunctional composite disc combining doxycycline-loaded and SPION-loaded microfiber was fabricated to integrate localized hyperthermia and antibiotic delivery within a single material.

## 2 Methods

### 2.1 Synthesis of magnetic nanoparticles

Undoped (γ-Fe_2_O_3_) and manganese-doped SPIONs (Mn_0.25_Fe_2.75_O_4_) were produced by flame spray pyrolysis (FSP) [38]. A liquid precursor solution was prepared by dissolving either iron(III) nitrate nonahydrate (purity 98 %; Sigma-Aldrich Sweden) alone or with manganese(II) nitrate tetrahydrate (purity 97 %; Sigma-Aldrich) in a solvent mixture (1:1) of 2-ethylhexanoic acid (98 %; Sigma-Aldrich) and ethanol (>99.7 %, HPLC grade; VWR, Belgium) to obtain a total metal concentration of 0.7 M. After stirring the solution for 1 h at room temperature, the precursor was fed at 6 mL min^−1^ and dispersed using 3 L min^−1^ O_2_ (>99.5 %, Linde AGA Gas AB, Sweden) at a constant pressure drop (1.6 bar). The flame was ignited by a supporting flame of premixed CH_4_ and O_2_ (>99.5 %, Linde AGA Gas AB) at flow rates of 1.5 and 3.2 L min^−1^, respectively. Gas flow rates were controlled with calibrated mass flow controllers (Bronkhorst, the Netherlands). The particles were collected on a glass fiber filter (Albert LabScience, Germany) with the aid of a Mink MM 1144 BV vacuum pump (Busch, Sweden).

### 2.2 Characterization of SPIONs

X-ray diffraction (XRD) patterns were recorded on a D2 PHASER X-ray diffractometer (Bruker, USA) equipped with Cu Kα radiation (λ = 1.5418 Å) at 30 kV and 10 mA using a step size of 0.02° 2θ and a scan speed of 2.00 deg min^−1^. The XRD patterns were analyzed by the Diffrac Eva 6.0 and TOPAS 4.2 software, and the mean crystallite size (d_XRD_) was calculated by Rietveld refinement and the Scherrer equation using the (311) plane. All patterns were normalized relative to the intensity of the peak corresponding to the (311) plane. Particle morphology was visualized by transmission electron microscopy (TEM) with a JEOL JEM-2100F equipment (Jeol Ltd., Japan) operating at 200 kV. The nanoparticle powder was suspended in 99.5 % ethanol and deposited as a 5 μL drop on a Formvar/Carbon 300 square mesh copper grid (Delta Microscopies, France). Sizes of 65 particles were measured using ImageJ software and plotted as histograms using the Sturges method [39]. The Shapiro-Wilk test confirmed that the size distributions of the sample followed a log-normal distribution, which was then fitted accordingly. The primary particle size was calculated as the geometric mean derived from the log-normal curve fitting.

The heat generated by SPIONs was measured using an alternating magnetic field (AMF) apparatus (magneTherm; Nanotherics Ltd., UK). For that purpose, first a 3 mg mL^−1^ aqueous suspension of SPIONs was prepared by sonication for 5 min at 90 % amplitude using a cup horn ultrasonicator (Sonics, USA), supplemented with a 10 s vortex mixing every 1 min. One mL of the nanoparticle suspension was transferred to a 2 mL glass vial and placed inside a 9-turn coil. The nominal oscillation frequency was set to 592 kHz and the magnetic field strength to 14 mT. The temperature of the suspension was measured with a fiber optic probe every second for 5 min. The specific absorption rate (SAR) was then calculated using equation (1).

(1) $$SAR=\frac{C_{p}m_{s}}{m_{n}}\times \frac{\mathrm{d}T}{\mathrm{d}t}$$

where, *m*_*s*_ and *C*_*p*_ are the mass and specific heat capacity of the suspension, which is approximated to the mass and specific heat capacity of water (1 g, 4180 J kg^−1^ K^−1^), *m*_*n*_ is the mass of nanoparticles, and *dT/dt* is the initial slope of the heating curve in the first 10 s [40].

### 2.3 Preparation of polymer mixtures for microfiber synthesis

To produce the PMMA microfibers, poly(methyl methacrylate) (PMMA, 120000 g mol^−1^) (Sigma-Aldrich) was dissolved in N,N-Di-methylformamide (DMF) (>99.5 %, HPLC grade; Thermo Scientific, USA) by stirring the mixture for 24 h at room temperature, at concentrations of 15, 20, 25, 27.5, and 30 wt% PMMA relative to final solutions. To produce microfibers with varying plasticizer content, PMMA and tributyl citrate (<99 %, Sigma-Aldrich) were dissolved in DMF to obtain solutions containing 27.5 wt% PMMA (relative to solution) and 0, 15, and 20 wt% tributyl citrate (relative to fiber weight). For synthesis of SPION-loaded microfibers containing 23 wt% SPIONs, a PMMA concentration of 25.4 wt% was used to obtain the same PMMA to DMF ratio as used for electrospinning 27.5 wt% PMMA solution. The polymer-SPION mixture was prepared by initially suspending the SPIONs and tributyl citrate (15 wt% relative to fiber weight) in DMF through vortex mixing for 10 s, followed by ultrasonication for 5 min at 90 % amplitude. The vortex mixing and sonication were repeated three times. Subsequently, PMMA was added to the SPION-tributyl citrate suspension and magnetically stirred for 24 h. The polymer solution for poly(lactic-co-glycolic acid) (PLGA, Resomer 503, lactide:glycolide 50:50, Sigma-Aldrich) fibers was prepared by dissolving 14 wt% of the polymer in 1,1,1,-3,3,3-hexa-fluoro-2-propanol (HFIP, Sigma-Aldrich), followed by overnight stirring. To prepare doxycycline-loaded PLGA fibers, 3.5 wt% of the drug was added to the already prepared PLGA solution and stirred for another hour.

### 2.4 Viscosity of the polymer mixtures

The viscosity measurements were performed using an AresG2 rheometer (TA Instruments, New Castle, DE, USA) equipped with a stainless-steel parallel plate geometry (25 mm diameter 0.0174 rad cone plate geometry). The measurements were carried out at 25 °C with flow sweep tests at shear rates between 0 and 30 s^−1^ (0.027 mm truncation gap, 244462 Pa N^−1^ m^−1^ stress constant, 57.4713 rad^-1^ strain constant, 4074.37 Pa N^−1^ normal stress constant).

### 2.5 Synthesis of microfibers

All microfibers were produced by electrospinning using a Fluidnatek LE-50 (Bioinicia, Spain) under controlled conditions of 25 ± 1 °C and 29 ± 5 % relative humidity. Electrospinning parameters used for various polymer mixtures are detailed in Table S1. The polymer solutions were fed through a 5 mL glass syringe, equipped with a No. 16/22 gauge needle. The procedure involved maintaining the emitter-to-collector distance of 14 cm, feed rate of 1–4 mL h^−1^ and an applied voltage of 8.6–14.2 kV, to achieve a stable Taylor cone. The fibers were deposited on the collector covered with aluminum foil, rotating at a speed of 200 rpm. The emitter scanned over a horizontal distance of 5 cm with a speed of 50 mm s^−1^. The rotating collector and horizontal scanning were performed to ensure homogenous fiber samples. The fibers were then carefully detached from the aluminum foil using a spatula and cut into desired shape.

### 2.6 Compaction of electrospun microfibers

To compress the microfibers into a compact material, fibers from different batches were stacked between two layers of aluminum foil or parchment paper, and placed between two copper plates (10 × 10 × 1 cm). The weight of the final compacted disc (measured with Mitutoyo QuantuMike IP65 Digital Micrometer) was controlled by controlling the number of stacked layers of fibers. The top copper plate weighed approximately 1.1 kg, while the bottom weighed 0.9 kg. Subsequently, this assembly was placed in a vacuum heating oven (Vacutherm VT 6025; Thermo Scientific, USA) for 1 h at a temperature of 90 °C for tributyl citrate containing microfibers, and at 130 °C for pure PMMA microfibers. PLGA and doxycycline-loaded PLGA microfiber discs were compacted at a temperature of 45 °C and 65 °C, respectively. The composite multifunctional microfiber discs were fabricated by compressing doxycycline-loaded PLGA fibers on top of pre-compacted magnetic microfibers at 65 °C.

### 2.7 Characterization of microfibers

Microfiber morphology was investigated using scanning electron microscopy (SEM) with the LEO 1530 system (Zeiss, Germany) with a voltage of 2 kV. To prevent charging of the polymer during SEM measurements, the microfibers were sputter coated with a 4 nm gold and palladium layer at 2 kV and 20 mA for 80 s using a SC7640 Auto/Manual High-Resolution Sputter Coater (Quorum Technologies, UK). The mean microfiber diameters of the fibers were determined by measuring 70–100 microfibers in ImageJ Software (version 1.53e, U.S. National Institutes of Health, Bethesda, MD, USA) [41]. The arithmetic mean and standard deviation were calculated from the measured fiber diameters. Chemical composition of microfibers and bulk materials was determined through attenuated total reflectance Fourier transform-infrared (FTIR) spectroscopy performed using an ALPHA II ATR-FTIR spectrometer (Bruker, USA). A total of 128 spectral scans were repeated over the range of 400–4000 cm^−1^. The X-ray diffraction (XRD) patterns of the microfibers were obtained using a D2 PHASER X-ray diffractometer (Bruker), employing identical instrument settings as those utilized for measuring the SPIONs.

Thermogravimetric analysis (Discovery TGA 550, TA Instruments, USA) was used to confirm the nanoparticle content in the SPION-loaded microfibers. Approximately 1.9 mg of SPION-loaded microfiber was placed on a platinum crucible and subjected to a temperature ramp from room temperature to 600 °C with a heating rate of 10 °C min^−1^, under nitrogen gas purge of 20 mL min^−1^. Mass losses were calculated from the obtained thermal curves normalized at 120 °C to exclude moisture adsorbed onto the microfibers. SPION content was calculated from initial fiber weight and the total inorganic mass remaining at the end of the measurement. Thermal behavior of the fibers was investigated using differential scanning calorimetry (DSC), performed using the Q1000 (TA Instruments, USA) under nitrogen gas flow of 20 mL min^−1^. Approximately 2–4 mg samples were placed in aluminum crucibles and the temperature was ramped from −20 °C to 200 °C, with a heating rate of 10 °C min^−1^. The mechanical properties of the electrospun fibrous discs were analyzed with a uniaxial tensile test using a texture analyzer (TA.XT plusC, Stable Micro System). Compacted microfibers of approximately 420 μm thickness (n = 3) were cut into a dog-bone shape (Fig. S1). The upper grip was moved with a speed of 0.02 mm s^−1^. The Young's modulus, ultimate tensile strength, and the elongation at break were calculated according to the stress-strain curves.

The release of SPIONs from the fibers was investigated by inductively coupled plasma optical emission spectroscopy (ICP-OES). Microfiber discs (14.1 mg each) were incubated in 500 μL of phosphate buffered saline (PBS) or culture medium (Luria broth, LB) for 40 min at temperatures of 37 °C or 55 °C. A control sample was prepared by soaking the microfiber sample (14.1 mg) in 500 μL mL HCl at room temperature and shaking it for approximately 56 h to release and dissolve the SPIONs from the microfiber. After incubation, 100 μL of the supernatant was collected from each incubation solution and mixed with 100 μL HCl. All solutions were further diluted with 5 % HNO_3_, filtered through 0.45 μm filters, and analyzed using ICP-OES. The fibers incubated in PBS and LB were later imaged using SEM to assess their morphological stability.

The magnetic properties of the microfibers and SPIONs were recorded on a vibrating sample magnetometer (Lake Shore, USA). Approximately 1 mg of Mn_0.25_Fe_2.75_O_4_ powder was used for the measurement, while the microfibers were analyzed using a 10 × 10 mm (1.9 mg) square piece of compacted SPION-loaded microfiber mat. Magnetization versus magnetic field was measured in the field range of ±1000 mT at room temperature. The magnetization curve of microfiber was normalized to the total SPION content.

To determine the heating efficiency of SPION-loaded microfibers, circular discs of 8 mm diameter were cut from the compacted microfiber mats. The discs were subjected to AMF in air using magneTherm (Nanotherics Ltd., UK). These discs were placed on a 3D-printed holder, placed in the center of a 9-turn coil, and exposed to AMF (14 mT, 592 kHz). The temperature was monitored using an infrared thermal camera (Fluke Ti480 Pro, Fluke Europe, the Netherlands). The samples underwent a 2 min equilibration to the temperature inside the coil, followed by a 10 min exposure to the AMF, and concluded with a 2 min cooling down period. An image was captured every 10 s over the 14 min period. The measurements were conducted in air using dry fibers and in PBS. The images were analyzed using SmartView 4.3 (Fluke Europe, the Netherlands) and a MATLAB 9.9 (MathWorks, Inc, USA) in-house script (https://github.com/MagnetFiber/Magnetic-Heating.git).

### 2.8 Antibacterial studies

Liquid cultures for antibacterial studies were prepared by inoculating MRSA (ST8: USA 300) in LB and incubating overnight at 37 °C at 220 rpm. The cultures were then adjusted to 10^5^ CFU mL^−1^ in LB. To determine the non-inhibitory concentration (NIC) and the minimum inhibitory concentration (MIC) of doxycycline, 200 μL of bacterial culture was incubated with varying concentrations of the antibiotic in honeycomb multiwell plates for 18 h. The tested doxycycline concentrations were 0.01, 0.1, 0.15, 0.25, 0.5, 1, 2, 4, 8, and 10 μg mL^1^. The plates were maintained at 37 °C with continuous shaking, and optical density at 600 nm (OD_600_) was measured after 18 h using a Bioscreen C instrument (Growth Curves OY, Turku, Finland). The percentage of bacterial growth relative to untreated control was plotted against the log_10_ of the antibiotic concentration, and a lognormal curve was fitted to the data. The NIC and MIC were then calculated using the Gompertz equation [42].

To analyze the synergistic effect of doxycycline (doxycycline hyclate, >93.5 %; Sigma-Aldrich) and heat, bacterial suspensions in LB were heated in a heat block (Techne Dri-Block heater) at temperatures of 45–60 °C for durations of 5–15 min. Following the heat treatment, 100 μL of bacterial suspensions was diluted with 100 μL of LB medium and mixed with a concentrated solution of doxycycline to achieve final concentrations of 0–0.15 μg ml^−1^. Untreated bacterial suspensions were obtained without the treatment of antibiotic or heat. After mixing, 200 μL of each bacterial suspension was transferred to honeycomb multiwell plates, which were incubated at 37 °C under constant shaking. The OD_600_ was measured every 10 min for 24 h using the Bioscreen C instrument. Growth inhibition was calculated by dividing the OD_600_ of the treated samples after 18 h by that of the untreated sample. Possible synergistic or additional effects were analyzed using the online tool SynergyFinder Version 3.0. The synergistic score was calculated using the Bliss model, which is based on the multiplicative effect of single drugs as if they acted independently [43,44].

The synergistic antibacterial efficacy of the SPION-loaded microfibers and doxycycline was evaluated using 8 mm (Ø) microfiber discs. Discs used for magnetic hyperthermia had an average weight of 15.6 ± 0.5 mg, while those used in non-heating experiments weighed approximately 12–16 mg. A narrow weight range was selected for hyperthermia studies to ensure reproducibility. The discs were first sterilized by UV light, with each side exposed for 1 h. Subsequently, the discs were placed in a sterile 7-well 3D printed holder, with each well containing 100 μL of MRSA bacterial suspension (10^6^ CFU mL^−1^) in LB. Freshly prepared doxycycline of different concentrations (0–0.5 μg mL^−1^) was added to the wells. The holders were incubated in a Carbolite Gero CWF 1300 furnace for 7 min at 60 °C to preheat the suspensions to 38 ± 2 °C. Thereafter, the holders containing microfiber discs were placed in a 9-turn coil and subjected to AMF (14 mT, 592 kHz) for 40 min to ensure at least 15 min of high-temperature (50–55 °C) exposure of the bacterial culture, followed by an incubation period of 18 h at 37 °C. Post-incubation, 100 μL of LB was added to each well, and bacterial samples were collected, centrifuged at 14000 × *g* for 2 min, and resuspended in 150 μL of LB to eliminate residual doxycycline. Subsequently, a ten-fold dilution sequence was prepared and 10 μL of the dilutions were spotted on Luria agar plates. The plates were then incubated at 37 °C for 16 h, followed by imaging and quantification of bacterial colonies.

### 2.9 Statistical analysis

Data analysis was performed using GraphPad Prism 10.0 software (La Jolla, CA, USA). Coefficient of determination (R^2^) was obtained from simple linear regression. Paired t-tests were used to compare the mechanical properties of microfibers. A two-way analysis of variance (ANOVA) using Tukey's multiple comparison test was used to compare different groups in antibacterial studies. *p* values were calculated as >0.05 (ns), ≤0.05 (∗), ≤0.01 (∗∗), ≤0.001 (∗∗∗), and ≤0.0001 (∗∗∗∗).

## 3 Results and discussion

### 3.1 Synthesis and characterization of flame-made SPIONs

Our previous study identified doped ferrites as high-performance SPIONs for magnetic hyperthermia with good biocompatibility [45]. Specifically, Mn-doped SPIONs showed better heating performance and lower cytotoxicity than undoped SPIONs, and were thus used in this study. The Mn_0.25_Fe_2.75_O_4_ and γ-Fe_2_O_3_ nanoparticles were synthesized by FSP (Fig. 1a). The morphological analysis of Mn-doped SPIONs through transmission electron microscopy (TEM) revealed a hexagonal particle shape, in excellent agreement with the literature (Fig. S2a) [38,46]. The average particle size (d_TEM_) of Mn-doped SPIONs determined from the TEM analysis was 17.7 nm. The geometric standard deviation (σ_g_) was 1.47, which corresponds to the theoretical self-preserving size distribution that is typically attained during flame synthesis (Fig. S2c) [37,47]. The X-ray diffraction (XRD) pattern of the Mn-doped SPIONs showed prominent diffraction peaks corresponding to the cubic spinel structure of maghemite or magnetite from the (111), (220), (311), (400), (422), (511), and (440) crystallographic planes (Fig. S2d) [38,46]. No peak corresponding to manganese oxide was observed. Previous studies have shown that during flame synthesis of SPIONs, the presence of a dopant in the iron oxide crystal lattice can lead to the formation of a magnetite phase [45,48]. The slight shift toward lower angles of the peaks in Mn-doped SPIONs compared to undoped SPIONs indicates the successful incorporation of Mn^2+^ ions into the iron oxide crystal [49,50]. This shift is a result of the larger ionic radius of Mn^2+^ ions (0.89 Å) compared to Fe^3+^ (0.64 Å), causing the lattice to expand [45,51]. The average crystallite size (d_XRD_) of Mn-doped SPIONs was 16 nm. This is in good agreement with d_TEM_, indicating predominantly monocrystalline particles. Furthermore, clear lattice fringes in TEM images (Fig. S2b) supported the existence of well-defined single crystalline particles showing the (111), (311), and (400) planes.

The magnetic hyperthermia efficiency of aqueous suspensions of Mn-doped SPIONs was assessed by measuring the heat released in an alternating magnetic field (AMF). The Mn_0.25_Fe_2.75_O_4_ nanoparticles exhibited a specific absorption rate of 113.4 W g^−1^, almost twice that of γ-Fe_2_O_3_ nanoparticles (67.6 W g^−1^). This is in agreement with the literature, confirming the superior heating capability of Mn-doped SPIONs compared to undoped SPIONs [38,45]. Therefore, Mn_0.25_Fe_2.75_O_4_ nanoparticles were used to produce magnetic microfibers in this study, and are henceforth referred to simply as SPIONs.

### 3.2 Synthesis and characterization of electrospun PMMA microfibers

**Fig. 2:**
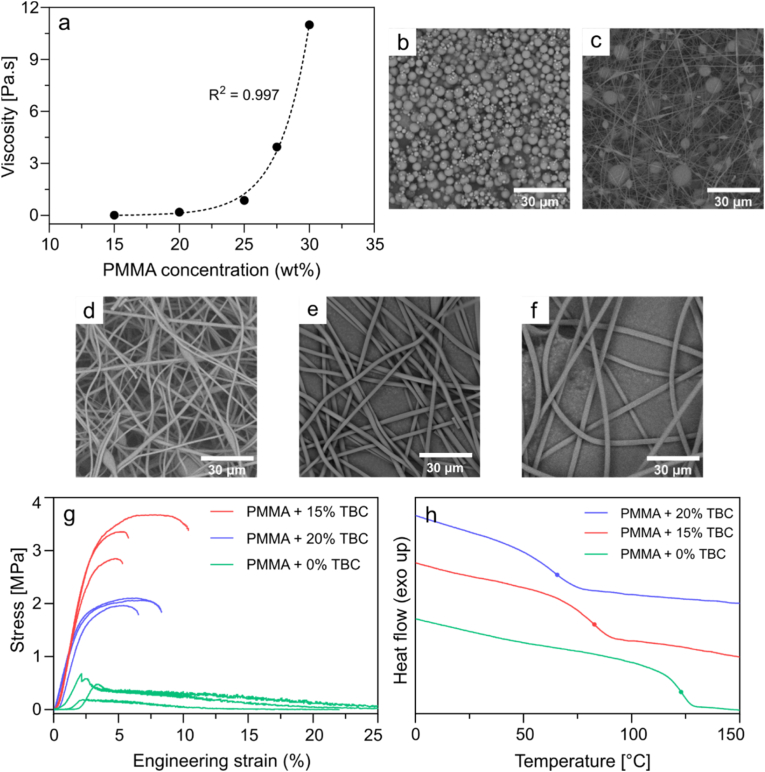
Characterization of electrospinning solution and PMMA microfibers. (a) Effect of PMMA concentration on viscosity of the solution at a shear rate of 30 s^−1^. The dotted line represents the exponential fit applied to the data, obtained using least squares regression. (b–f) SEM images of electrospun fibers with PMMA concentration of (b) 15 wt%, (c) 20 wt%, (d) 25 wt%, (e) 27.5 wt%, and (f) 30 wt%. (g) Stress-strain curves of PMMA microfibers with 0, 15, and 20 wt% tributyl citrate (TBC). (h) DSC thermograms of PMMA microfibers with 0, 15, and 20 wt% TBC. The dots represent the midpoint glass transition temperature (*T*_g_) at 123, 82 and 65 °C, respectively.

PMMA is a rigid polymer, widely used for fabrication of microfibers for biomedical applications, with demonstrated biocompatibility in both *in vitro* and *in vivo* studies [23,52]. Furthermore, PMMA is approved by the FDA for use in medical devices [53]. SPION-loaded PMMA has been successfully used in bone cement and nanofibrous composites for magnetic hyperthermia therapy [33,54]. Therefore, PMMA was used in this study to fabricate magnetic microfibers through electrospinning (Fig. 1b). First, electrospinning of pure PMMA microfibers was investigated using different PMMA concentrations (15, 20, 25, 27.5, and 30 wt%) (Table S1). The effect of PMMA concentration on the viscosity of polymer solution is shown in Fig. 2a. Increasing the PMMA concentration from 15 wt% to 30 wt%, exponentially increased the mixture viscosity from 0.02 Pa⋅s to 11 Pa⋅s. The scanning electron microscopy (SEM) image (Fig. 2b) of the product obtained from electrospinning a solution of 15 wt% PMMA shows the formation of microspheres. These microspheres are likely formed due to the electrospinning jet breaking up into droplets, a phenomenon recognized as electro-spraying [55]. With an increase in polymer concentration to 20 wt%, the morphology transformed into non-uniform spindle-like fibers, still with prominent beads (Fig. 2c). After exceeding a polymer concentration threshold necessary for uniform fiber production, an increase in polymer content led to an increase in fiber diameter (Fig. S3), which is in agreement with previous studies [56,57].

The SEM image of fibers produced using 25 wt% PMMA (Fig. 2d) shows reduced bead frequency. The change in morphology compared to fibers with lower PMMA concentrations could be due to the higher surface tension and lower solvent content in solutions with higher PMMA concentrations [57]. Increasing the PMMA concentration to 27.5 wt% resulted in bead-free and randomly oriented fibers (Fig. 2e). The highest polymer concentration tested (30 wt%) also resulted in the production of continuous beadless fibers with a smooth surface (Fig. 2f). However, the high viscosity of the 30 wt% PMMA solution (11 Pa⋅s) made the solution preparation and handling challenging. Therefore, to mitigate bead formation and maintain relatively low viscosity of the polymer solution, a PMMA concentration of 27.5 wt% was selected for subsequent experiments.

The microfibers produced using just PMMA were brittle and broke apart during manual handling. In order to reduce the brittleness of the microfibers, tributyl citrate was introduced as a plasticizer into the electrospinning solution of 27.5 wt% PMMA [58], and tested at varying concentrations of 0, 15, and 20 wt%, with respect to the microfiber weight. Regardless of the tributyl citrate content, all compositions produced fibers with smooth surfaces, displaying minimal to no beads-on-a-string morphologies (Fig. S4). To measure the tensile properties of microfibers, they were first compressed into a compact material by stacking them. The stress-strain curves (Fig. 2g) of microfibers at different tributyl citrate concentrations were measured using a dog-bone shaped cutout of the compacted microfibers (Fig. S1). Table S2 summarizes the mechanical properties for various microfiber compositions. The pure PMMA microfibers show the lowest ultimate tensile strength at 0.36 ± 0.23 MPa, indicative of their inherent brittleness. Incorporation of 15 wt% tributyl citrate enhances stress tolerance significantly, resulting in a peak tensile strength of 3.29 ± 0.34 MPa. This composition allows for some plastic deformation, evident by a strain of 7.2 ± 2.3 % at fracture, showing improved flexibility. Interestingly, further increase in tributyl citrate concentration to 20 wt% led to a reduction in ultimate tensile strength. This suggests a critical concentration threshold beyond which excessive plasticization may compromise mechanical integrity [59].

The thermal properties of the microfibers were analyzed using differential scanning calorimetry (DSC) (Fig. 2h). The glass transition temperature (*T*_g_) of the microfibers decreased from 123 °C at 0 wt% tributyl citrate, to 82 and 65 °C at 15 and 20 wt% tributyl citrate, respectively. The reduction in *T*_g_ indicates a transition from a glassy and rigid to a more flexible state, caused by reduced polymer–polymer interactions and increased chain mobility [60]. Magnetic hyperthermia treatment for MRSA requires temperatures close to 55 °C. However, a *T*_g_ of 65 °C observed with 20 wt% tributyl citrate is too close to the application temperature, which can compromise microfiber integrity due to softening and reduced mechanical stability. The 15 wt% tributyl citrate with a higher *T*_g_ of 82 °C offers both the necessary tensile strength and thermal stability for the microfibers required during hyperthermia treatment. Consequently, PMMA microfibers containing 15 wt% tributyl citrate were used in subsequent experiments.

### 3.3 Synthesis and characterization of magnetic microfibers

The magnetic microfibers were prepared by incorporating 23 wt% SPIONs and 15 wt% tributyl citrate (relative to the fiber weight), into the electrospinning mixture containing 25.5 wt% PMMA. Demir et al. previously showed that incorporating more than 24 wt% SPIONs into poly(ε-caprolactone) resulted in bead formation and structural irregularities in electrospun microfibers [61]. To minimize such defects in the PMMA fibers while maximizing magnetic heating performance, a SPION content of 23 wt% was selected as an optimal compromise between electrospinning processability and magnetic microfiber performance. The PMMA concentration was adjusted from 27.5 wt% (Fig. 2) to 25.4 wt% in the current formulation. This change was made to maintain a consistent PMMA-to-solvent ratio of 27.5 wt%, ensuring sufficient viscosity for successful processing (Table S1). The resulting microfibers containing magnetic nanoparticles are referred to as SPION-loaded microfibers. SPION content in these fibers was confirmed using thermogravimetric analysis. The non-loaded microfibers were prepared as controls, containing only PMMA and tributyl citrate (15 wt%).

**Fig. 3:**
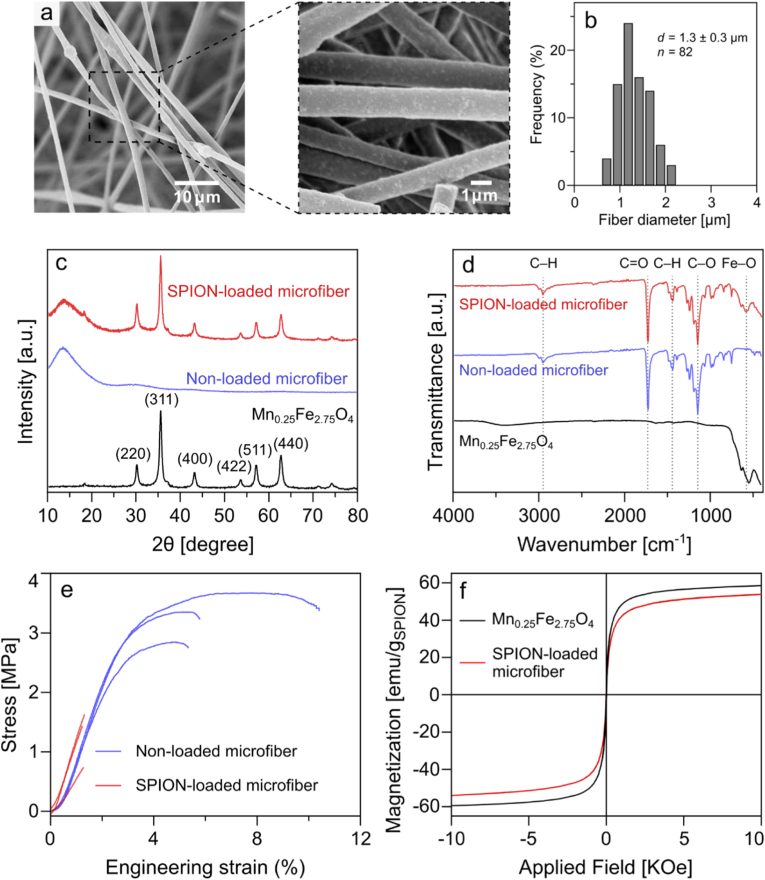
Structural, chemical, mechanical, and magnetic properties of SPION-loaded microfibers. (a) SEM images and (b) fiber diameter distribution of SPION-loaded microfibers. (c) XRD patterns and (d) FTIR spectra of SPION-loaded microfibers, non-loaded microfibers, and Mn_0.25_Fe_2.75_O_4_ nanoparticles. (e) Stress-strain curves of SPION-loaded and non-loaded microfibers. The curves end at the fiber fracture point of the SPION-loaded microfibers. (f) Magnetization curves of SPION-loaded microfibers and Mn_0.25_Fe_2.75_O_4_ nanoparticles.

The SEM images of SPION-loaded microfibers (Fig. 3a) displayed a rougher surface compared to the control (Fig. S4b), and had an average fiber diameter of 1.3 ± 0.3 μm (Fig. 3b). The SPION-loaded microfibers were characterized by XRD (Fig. 3c), along with pure PMMA microfiber and Mn_0.25_Fe_2.75_O_4_ nanoparticles for comparison. The SPION-loaded microfibers show sharp characteristic peaks of the Mn_0.25_Fe_2.75_O_4_ at the (220), (311), (400), (422), (511), and (440) crystallographic planes, indicating that the nanoparticles maintain their crystal structure after incorporation into the microfiber. The broad hump observed around 2θ of 14° can be attributed to amorphous PMMA polymer. The chemical composition of the microfibers was further assessed by Fourier-transform infrared spectroscopy (FTIR) (Fig. 3d). The spectra for SPION-loaded microfibers show peaks at 2952 and 1444 cm^−1^ which can be attributed to C–H stretching and C–H bending modes, respectively [62]. These peaks demonstrate no discernible changes compared to those observed in the PMMA/tributyl citrate microfiber, suggesting that the C–H groups do not interact with the nanoparticles. The SPION-loaded microfibers also show peaks at 1730 cm^−1^ (C=O stretching), 1147 cm^−1^ (C–O vibrations), and 580 cm^−1^ (Fe–O vibration). The Fe–O peak in SPION-loaded microfibers exhibits a slight shift compared to Mn_0.25_Fe_2.75_O_4_ nanoparticles, implying intermolecular interaction between the polymer matrix and the nanoparticles [63]. The SPION-loaded microfibers displayed a broadened glass transition region, with slightly reduced *T*_g_ (76 °C) compared to non-loaded fibers (82 °C). This broad transition suggests increased heterogeneity in the polymer matrix, likely due to disruption in polymer chain packing and mobility caused by SPION loading.

The mechanical properties of the SPION-loaded microfibers were evaluated through the stress-strain curves shown in Fig. 3e. The introduction of nanoparticles into the polymer matrix did not significantly affect the Young's modulus of the microfibers (Table S2), indicating no change in material's stiffness and rigidity. However, the ultimate tensile strength decreased significantly from 3.3 MPa to 1.3 MPa, suggesting that the incorporation of nanoparticles weakens the microfibers leading to failure at lower stress. Furthermore, the strain at fracture decreased from 7.2 % to 1.2 %, indicating a reduction in the material's plasticity. The decrease in elongation at break of the SPION-loaded microfiber has been reported at high SPION loading (20 wt%) in PMMA microfibers, attributed to the reduction in fiber necking at increased particle content [64]. Overall, in this study the incorporation of SPIONs led to changes in the mechanical properties of the microfiber, decreasing its strength, and ductility. Nonetheless, the fibers retained sufficient mechanical integrity for their intended use.

The magnetic properties of SPION-loaded microfibers were analyzed using vibrating sample magnetometry and the magnetization curves are shown in Fig. 3f. The microfibers exhibit zero hysteresis indicating superparamagnetic behavior, signifying that the SPIONs retain their superparamagnetic property after incorporation into the polymer matrix. Furthermore, slight reduction in saturation magnetization is observed after incorporation of SPIONs into the microfibers, decreasing from 58.4 emu g_SPION_^−1^ to 53.7 emu g_SPION_^−1^. This change in saturation magnetization is likely due to shielding of the nanoparticle core by the polymer matrix, which has been previously reported for various types of coating materials [65].

The structural stability of magnetic microfibers and their SPION release were analyzed in PBS and culture medium (Luria broth) at both physiological (37 °C) and elevated (55 °C) temperatures after 40 min of incubation. SEM images showed no noticeable degradation of the microfiber discs under any of the conditions, indicating good material stability (Fig. S5). The release of SPIONs from the microfibers was further assessed using ICP-OES and compared to a control disc incubated in HCl for approximately 56 h. SPION release was consistently less than 0.05 % across all samples, with no noticeable differences observed between the various conditions. Moreover, the total concentration of released SPIONs was below 1 μg mL^−1^, >500 times lower than the reported cytocompatible concentration [45]. These findings demonstrate the stability of the microfibers and suggest their potential for safe biomedical applications. Overall, the SPION-loaded microfibers exhibit promising physicochemical, mechanical, and magnetic properties for antimicrobial applications.

### 3.4 Heating efficiency of magnetic microfibers

**Fig. 4:**
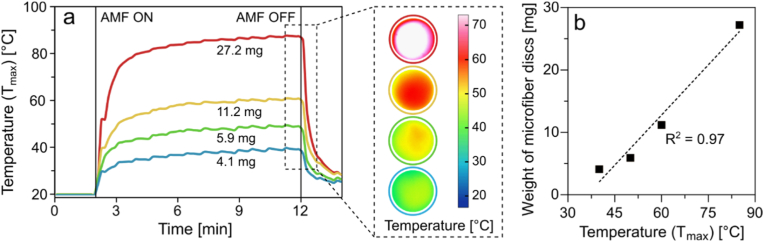
Heating efficiency of SPION-loaded microfibers in air. (a) Temperature of microfiber discs as a function of AMF exposure time for SPION-loaded microfibers of different weights. The measurements were performed in air using dry 8 mm (Ø) microfiber discs. AMF was turned on at t = 2 min and turned off at t = 12 min. The inset shows the infrared images of the different discs at t = 12 min. The color scale indicates the temperature of the fiber discs. (b) Effect of weight of SPION-loaded microfiber discs on the maximum temperature achieved in AMF. (For interpretation of the references to color in this figure legend, the reader is referred to the Web version of this article.)

The heating efficiency under an AMF was measured in microfibers of varying weights, which were formed by compacting layers of electrospun microfibers into a mat and cutting it into discs of 8 mm diameter (Fig. S6a and b). The heating profiles of these compacted microfiber discs were measured in air (Fig. 4a) at 14 mT and 592 kHz. The AMF parameters were selected based on recent comprehensive studies on clinically permissible AMF parameters [66,67]. The maximum temperature achieved by the discs increased with increase in their weight. The 4.1 mg disc heated to 40 °C, while the 27.2 mg disc reached a temperature of 84 °C. Across all discs, the temperature elevation occurred rapidly, achieving over 80 % of the maximum temperature within the first minute. The thermal images of the microfiber discs indicated nearly homogeneous heating (Fig. 4a inset), suggesting uniform distribution of SPIONs within the microfiber mat. Slightly higher temperatures were observed in the center of the microfiber discs, possibly due to cooling of the edges exposed to the surrounding environment. The maximum temperature achieved by the discs correlated strongly to their weight (Fig. 4b), likely due to the increase in the amount of SPIONs in the microfiber discs with increase in the disc weight. These findings emphasize the controllability of microfiber heating efficiency and the uniformity of heating across the disc area.

The magnetic heating of microfibers in the above experiments was conducted in air as the surrounding medium, relevant particularly for topical applications, such as the treatment of wound infections. However, to account for implant-associated therapies, we also investigated the heating efficacy in aqueous medium (PBS), which showed a relatively slower temperature increase (Fig. S6c). Magnetic heating in aqueous environments is more challenging due to the significantly higher heat capacity of water than air, allowing it to absorb heat more effectively. This makes it harder to sustain elevated temperatures in aqueous media, necessitating higher SPION content, prolonged AMF exposure, or stronger magnetic fields. Therefore, in subsequent experiments, discs weighing approximately 15.6 mg were used. The disc weight was based on the measurements in air, where temperatures of at least 60 °C were reached, which is critical for antibacterial efficacy without damaging the surrounding tissue [10]. Furthermore, the disc weight maintained the flexibility of the microfiber while preventing excessive thickness.

Preliminary experiments were conducted to assess the heating efficiency of these microfiber discs in culture medium, which indicated that at least 40 min of AMF exposure was required to sustain a high temperature (>50 °C) for 15 min.

### 3.5 Synergistic antibacterial efficacy of magnetic hyperthermia and antibiotic

**Fig. 5:**
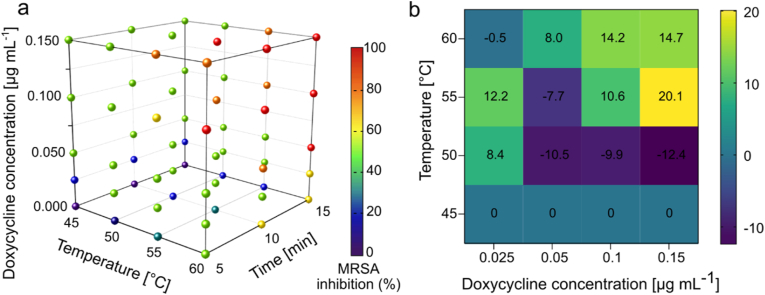
Antibacterial effect of heat and antibiotic on MRSA growth. (a) Growth inhibition of MRSA (measured after 18 h) following exposure to different temperatures (45–60 °C), for durations of 5, 10, and 15 min, in the presence of varying concentrations of doxycycline (0–0.15 μg mL^−1^). The color scale indicates the growth inhibition of MRSA based on OD_600_ measurements. (b) Synergy score, calculated using the BLISS model, illustrating the combined effect of a 15-min heat exposure and doxycycline on MRSA growth after 18 h of incubation. Triplicate measurements were analyzed using the SynergyFinder software. (For interpretation of the references to color in this figure legend, the reader is referred to the Web version of this article.)

The impact of combining heat with antibiotic treatment depends on factors such as the type of antibiotic, the applied temperature, and the bacterial strain [68,69]. To investigate these factors for MRSA, first we determined the non-inhibitory concentration (NIC) and the minimum inhibitory concentration (MIC) of doxycycline to be 0.05 μg mL^−1^ and 0.22 μg mL^−1^, respectively (Fig. S7a and b). Maintaining doxycycline concentrations below the MIC is essential to discern the contribution of hyperthermia to antibacterial efficacy. Thus, we investigated the effect of sub-inhibitory doxycycline concentrations (0–0.15 μg mL^−1^), elevated temperature (45–60 °C), and heating duration (5–15 min) on growth inhibition of MRSA by using heating blocks (Fig. 5a). Doxycycline is expected to remain stable at the temperatures applied in this study, which are well below its degradation threshold (>120 °C) [70,71]. The application of heat treatment alone at 45 °C did not yield any discernible effect, even with extended exposure time. This observation aligns with previous reports on photothermal antibacterial treatment, which demonstrated less than 1-log reduction of *S. aureus* biofilms after heating up to 45 °C [72], and a 10 % reduction in MRSA growth following treatment at 50 °C [13]. In this study, doxycycline treatment at the lowest dose of 0.025 μg mL^−1^ combined with heating at 45 °C, resulted in a 25 % reduction in bacterial growth. However, increasing the doxycycline concentration to 0.05 μg mL^−1^, significantly decreased MRSA growth by up to 50 %. Moreover, combining a 0.025 μg mL^−1^ dose of doxycycline with heat treatment at 60 °C for 15 min achieved 70 % inhibition of MRSA growth. The duration of exposure appeared to be pivotal in these experiments, with a 15 min exposure yielding significantly improved outcomes. Consequently, we selected a high-temperature exposure duration of at least 15 min for subsequent antibacterial *in vitro* assays.

The combined antibacterial effect of heating with doxycycline was investigated using the SynergyFinder (3.0) software [43]. The synergy scores for combinatorial treatments were obtained using the Bliss model (Fig. 5b). The values below −10 suggest an antagonistic effect, between −10 and 10 indicate an additive effect, while above 10 suggest a synergistic effect between the treatments. A synergy score near zero gives limited confidence in synergy or antagonism [43]. The synergy scores indicate that the doxycycline concentration of 0.025 μg mL^−1^, which is below the NIC of 0.05 μg mL^−1^ for MRSA (Fig. S7a), may not be sufficient to independently exert a significant antibacterial effect. However, when combined with heat treatment at 55 °C, the sub-NIC concentration likely sensitizes the bacteria, making them more susceptible to the drug. This combination results in a BLISS value of +12.2, indicating synergistic activity. Interestingly, we observed antagonism between hyperthermia and doxycycline at <55 °C. This may reflect a temperature threshold where sublethal heat stress induces bacterial adaptations that reduce antibiotic susceptibility [73,74]. Given the bacteriostatic nature of doxycycline, altered bacterial growth dynamics under heat stress could also play a role. However, this phenomenon is scarcely addressed in current literature and further studies are needed to investigate the underlying temperature-dependent interaction.

The ability of heat to enhance the effect of doxycycline at a sub-inhibitory dose is a key finding that highlights the potential of hyperthermia treatment. At an intermediate doxycycline concentration of 0.05 μg mL^−1^, the effect of drug alone may dominate much more than heat, resulting only in an additive effect. At higher concentrations of doxycycline, the bacteria may become more susceptible due to both the direct cytotoxic effects of the higher dose and the potentiating effects of heat. These findings agree with Ziesmer et al. who reported that vancomycin concentrations >0.5 μg mL^−1^ and heating to around 60 °C were necessary to achieve synergy against MRSA. They identified lower temperatures (45–55 °C) as an optimal therapeutic window to reduce adverse thermal effects [13]. Similarly, Kwan et al. showed successful synergistic treatment of *S. aureus* biofilms <50 °C without tissue harm [75]. Therefore, to enable synergistic action in our study and to minimize thermally induced side effects of high temperature, we used an AMF treatment that achieved temperatures below 55 °C [76], and a doxycycline concentration below the MIC (Fig. S7b). Future studies should explore underlying mechanisms, such as changes in bacterial membrane permeability following combined hyperthermia and antibiotic treatment [5].

**Fig. 6:**
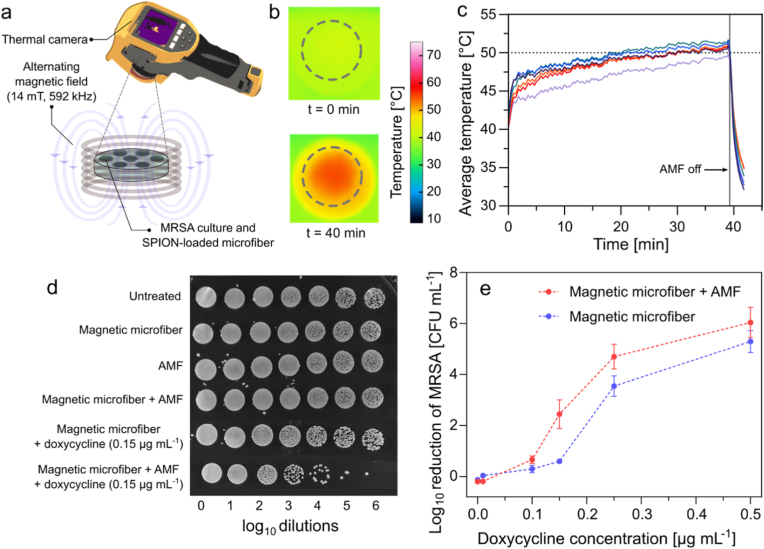
Synergistic antibacterial effect of magnetic hyperthermia and doxycycline against MRSA. (a) Schematic of the experimental setup showing 3D printed holder with six wells subjected to AMF (14 mT, 592 kHz) for approximately 40 min. The AMF was switched on at t = 0 min and switched off at t = 39.5 min. Each well contained SPION-loaded microfiber disc, MRSA culture, and doxycycline solution. (b) Representative thermal images of the disc at t = 0 min (top) and t = 39.5 min (bottom). (c) Average temperature in the measurement area of the six wells as a function of AMF exposure time. (d) Spot dilution assay showing 10-fold serial dilutions of MRSA cultures post treatment with SPION-loaded microfibers, doxycycline (0.15 μg mL^−1^), and AMF. Cultures were spotted following 18 h of incubation after treatment. (e) Reduction in MRSA colony forming units (CFUs) after treatment. Data represented as mean ± SD (n = 3).

The *in vitro* antibacterial effect of magnetic-microfiber induced hyperthermia and doxycycline was evaluated in MRSA cultures under clinically relevant AMF conditions (14 mT, 592 kHz) (Fig. 6a). Change in surface temperature on top of the wells was monitored using a thermal camera. The cultures were exposed to AMF for 40 min, to compensate for the slow temperature increase of microfibers in aqueous medium. Fig. 6b shows representative thermal images of the well at 0 min and 40 min. The average temperature within the measurement area (dotted circle, Fig. 6b) in the wells was measured as a function of time (Fig. 6c). The temperature of the wells gradually increased upon AMF exposure, and was maintained between 50 and 55 °C for 15 min. Doxycycline concentrations were varied between 0.01 and 0.5 μg mL^−1^ to include both sub-inhibitory and above-MIC concentrations.

Spot dilution assay was used to investigate the effect of the treatments (Fig. 6d and Fig. S8). The presence of SPION-loaded microfiber or AMF alone did not affect the growth of MRSA. Furthermore, magnetic hyperthermia without doxycycline did not considerably inhibit the growth of MRSA, suggesting that the temperature increase itself is not sufficient to induce bacterial inhibition. This finding is consistent with the results from the heating block experiments (Fig. 5a). However, combining doxycycline with hyperthermia significantly reduced the number of colony forming units (CFUs) (Fig. 6d). The growth inhibition of MRSA as a function of the combinatorial treatment is shown in Fig. 6e. A doxycycline dose of 0.15 μg mL^−1^ achieved a 0.59 log_10_ reduction in MRSA growth. However, when combined with magnetic hyperthermia, the reduction increased to 2.43 log_10_, representing a 35 % improvement in the efficacy of the treatment at sub-inhibitory doxycycline concentration. This enhancement in antibacterial efficacy was also observed at much higher doxycycline doses.

The magnetic microfiber mediated hyperthermia not only boosts localized bacterial growth reduction but also offers a strategy to minimize antibiotic dosage, potentially mitigating resistance development and adverse effects. Furthermore, the heating profiles can be finely tuned by adjusting the AMF parameters (field strength, duration), nanoparticle loading, and the microfiber density. Non-invasive thermometry methods, such as infrared thermal imaging, can ensure the safe application of this technology by maintaining temperatures within therapeutic ranges and minimizing off-target heating.

**Fig. 7:**
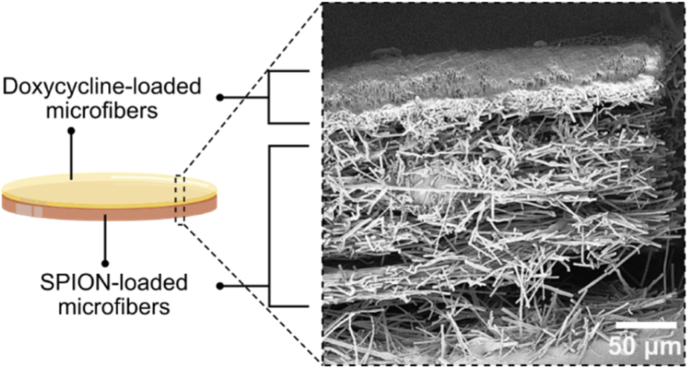
Illustration of multifunctional microfiber discs and its cross-sectional SEM image. The disc was composed of a top layer of doxycycline-loaded microfibers and a bottom layer of SPION-loaded microfibers.

Finally, to explore the therapeutic potential of the microfiber-based approach, we developed a proof-of-concept multifunctional platform combining drug-loaded and SPION-loaded microfibers. Doxycycline-loaded poly(lactic-co-glycolic acid) (PLGA) microfiber discs were fabricated, exhibiting uniform morphology, smooth surface, and successful drug incorporation (Fig. S9). These antibiotic-loaded fibers were then compacted together with SPION-loaded microfibers to form a multifunctional composite patch (Fig. 7). This multifunctional platform demonstrates the potential to integrate localized hyperthermia and antibiotic delivery within a single composite material. Future development of such multifunctional microfiber discs should include assessments in physiologically representative models, such as *ex vivo* infection systems [77], and *in vivo* studies [78], to enhance understanding of their cytocompatibility and therapeutic potential.

## 4 Conclusion

This study presents SPION-loaded magnetic microfibers designed for synergistic treatment of MRSA infections with antibiotics. SPION synthesis and microfiber fabrication were conducted using scalable methods, FSP and electrospinning, respectively. The fabrication of microfibers was optimized by investigating the impact of polymer composition on structural, chemical, thermal and tensile properties of the microfibers. The optimized SPION-loaded microfibers exhibited a uniform fiber structure with adequate tensile strength. On exposure to AMF, the microfiber discs reached temperatures exceeding 60 °C, with the flexibility to adjust hyperthermia temperatures by varying the microfiber disc weight. The tunable heating profile of microfibers allows adaptability to match thermal sensitivity of different bacteria-antibiotic combinations and also minimizes potential damage to surrounding tissues by preventing overheating. Furthermore, the combination of doxycycline at sub-inhibitory concentrations and heat demonstrated a synergistic effect on MRSA growth inhibition after 15 min of heating at 55 °C or higher. Finally, the application of magnetic hyperthermia significantly enhanced the efficacy of doxycycline against MRSA, showing a 35 % improvement at sub-inhibitory doxycycline concentrations. Importantly, this approach enabled the use of lower antibiotic dose, which is critical in combating antimicrobial resistance. The combination of SPION-loaded microfibers for magnetic hyperthermia with antibiotic therapy represents a significant advancement in local antibacterial treatment of resistant infections.

## CRediT authorship contribution statement

**Shaquib Rahman Ansari:** Writing – review & editing, Writing – original draft, Visualization, Methodology, Investigation, Formal analysis, Conceptualization. **Dominique Grimm:** Writing – review & editing, Writing – original draft, Investigation, Formal analysis. **Reshma V. Ramachandran:** Writing – review & editing, Methodology, Investigation, Formal analysis. **Yael del Carmen Suárez-López:** Writing – review & editing, Investigation. **Krisztina Juriga-Tóth:** Writing – review & editing, Investigation, Funding acquisition. **Georgios A. Sotiriou:** Writing – review & editing, Supervision, Resources, Methodology, Funding acquisition, Conceptualization. **Alexandra Teleki:** Writing – review & editing, Supervision, Resources, Project administration, Methodology, Funding acquisition, Conceptualization.

## Declaration of competing interest

The authors declare that they have no known competing financial interests or personal relationships that could have appeared to influence the work reported in this paper.

## Data Availability

Data will be made available on request.
