# Supplementary material for: Magnetic microfiber hyperthermia for synergistic antimicrobial activity against methicillin-resistant Staphylococcus aureus
Source: Mater Today Bio. 2025 May 12;32:101862. doi: 10.1016/j.mtbio.2025.101862 (PMC12141555; doi:10.1016/j.mtbio.2025.101862)
Supplement: Multimedia component 1 [file mmc1.docx]

Supporting Information

Magnetic microfiber hyperthermia for synergistic antimicrobial activity against methicillin-resistant *Staphylococcus aureus*

Shaquib Rahman Ansari^a^, Dominique Grimm^a^, Reshma V. Ramachandran^b^, Yael del Carmen Suárez-López^a^, Krisztina Juriga-Tóth^a^, Georgios A. Sotiriou^b,c^, and Alexandra Teleki^a,*^

^a^ Department of Pharmacy, Science for Life Laboratory, Uppsala University, 75123 Uppsala, Sweden
^b^ Department of Microbiology, Tumor and Cell Biology, Karolinska Institute, 17177 Stockholm, Sweden

^c^ Department of Chemistry, Science for Life Laboratory, Stockholm University, 11418 Stockholm, Sweden

^*^ Corresponding author, email: alexandra.teleki@scilifelab.uu.se


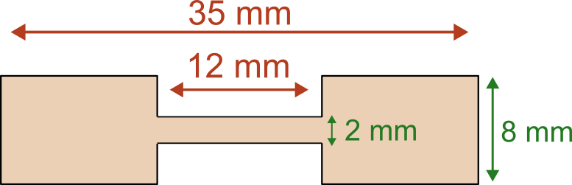


**Fig. S1.** Schematic of the dog-bone shaped cutout used to measure the mechanical properties of microfibers. Total length = 35 mm, total width = 8 mm, length of narrow portion = 12 mm, width of narrow portion = 2 mm.


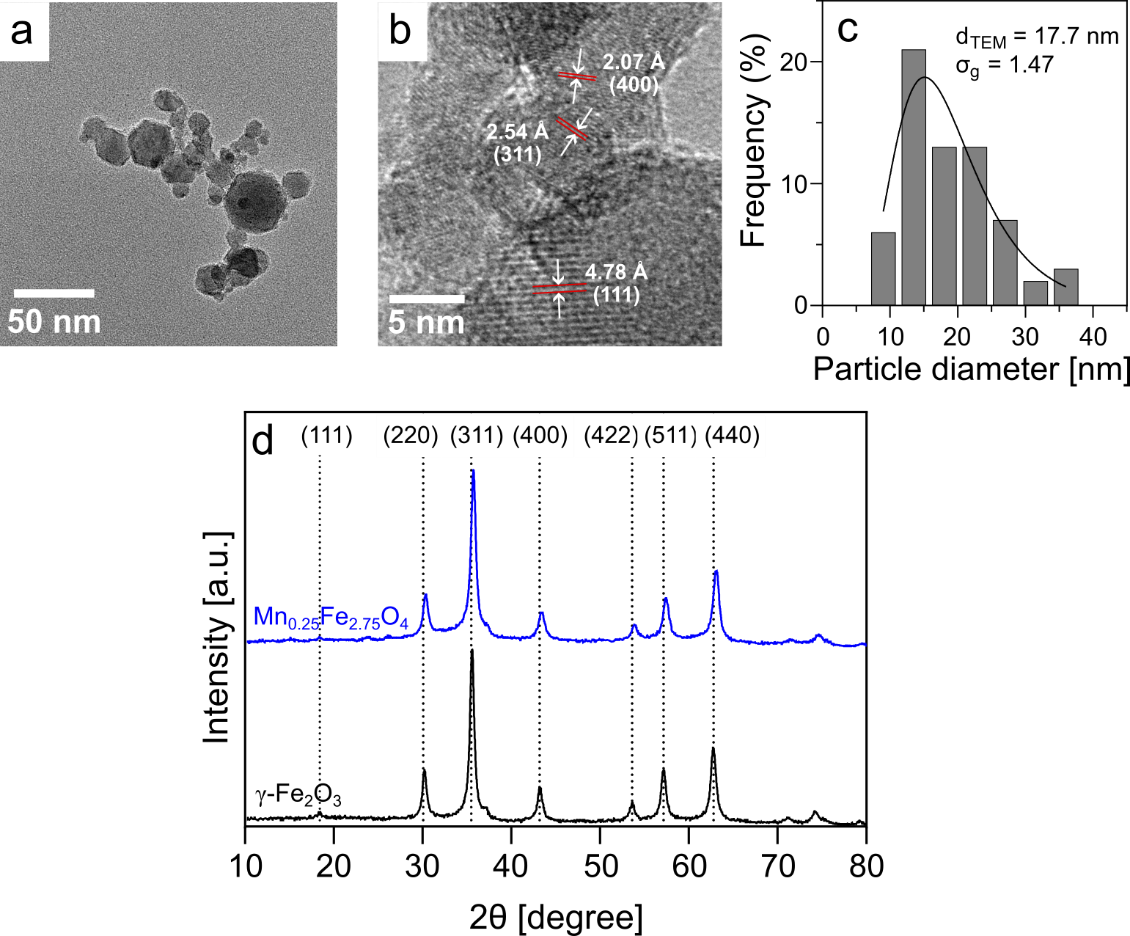


**Fig. S2.** Structural characterization of Mn_0.25_Fe_2.75_O_4_ nanoparticles. (a) TEM image, (b) high resolution TEM image showing the d-spacing with fringe separations of (400), (311), and (111) crystallographic planes, and (c) particle size distribution. The average particle size was determined by analyzing 65 particles. The solid line in (c) represents the log-normal fit of the histogram. (d) XRD patterns of γ‑Fe_2_O_3_ (black ) and Mn_0.25_Fe_2.75_O_4_ (blue) nanoparticles.


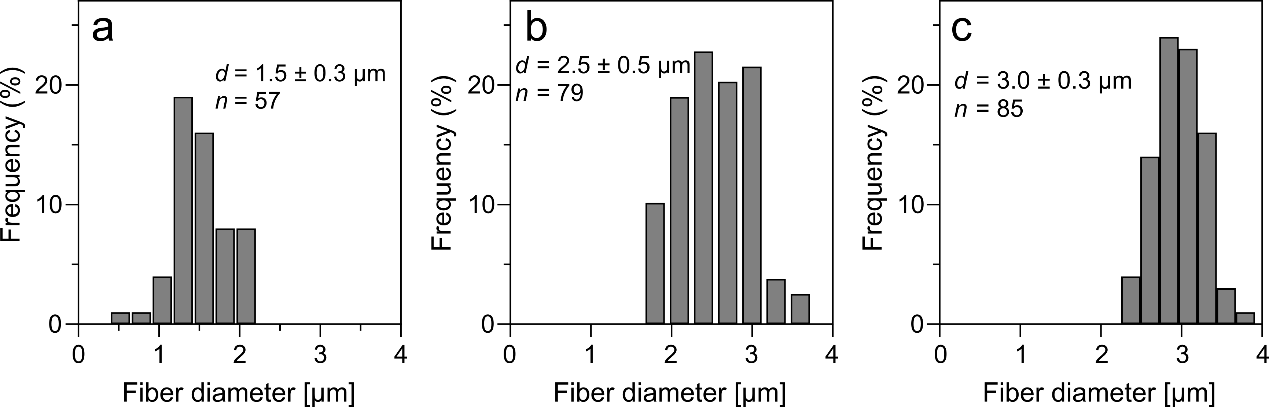


**Fig. S3.** Fiber diameter distribution of (a) 25 wt%, (b) 27.5 wt%, and (c) 30 wt% PMMA microfibers.


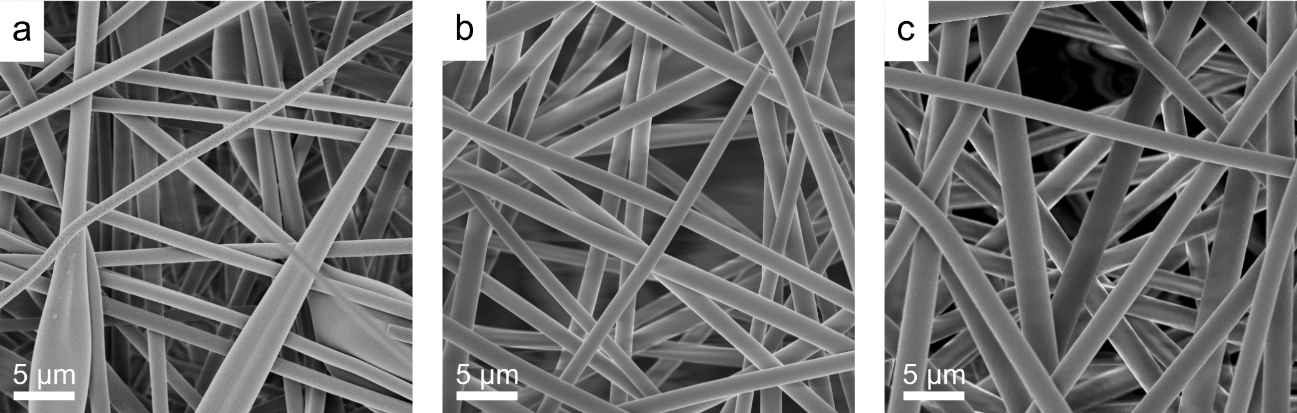


**Fig. S4.** SEM images of PMMA microfibers electrospun with tributyl citrate (TBC) concentrations of (a) 0 wt% (b) 15 wt%, and (c) 20 wt%. The TBC content is expressed relative to the final fiber weight.


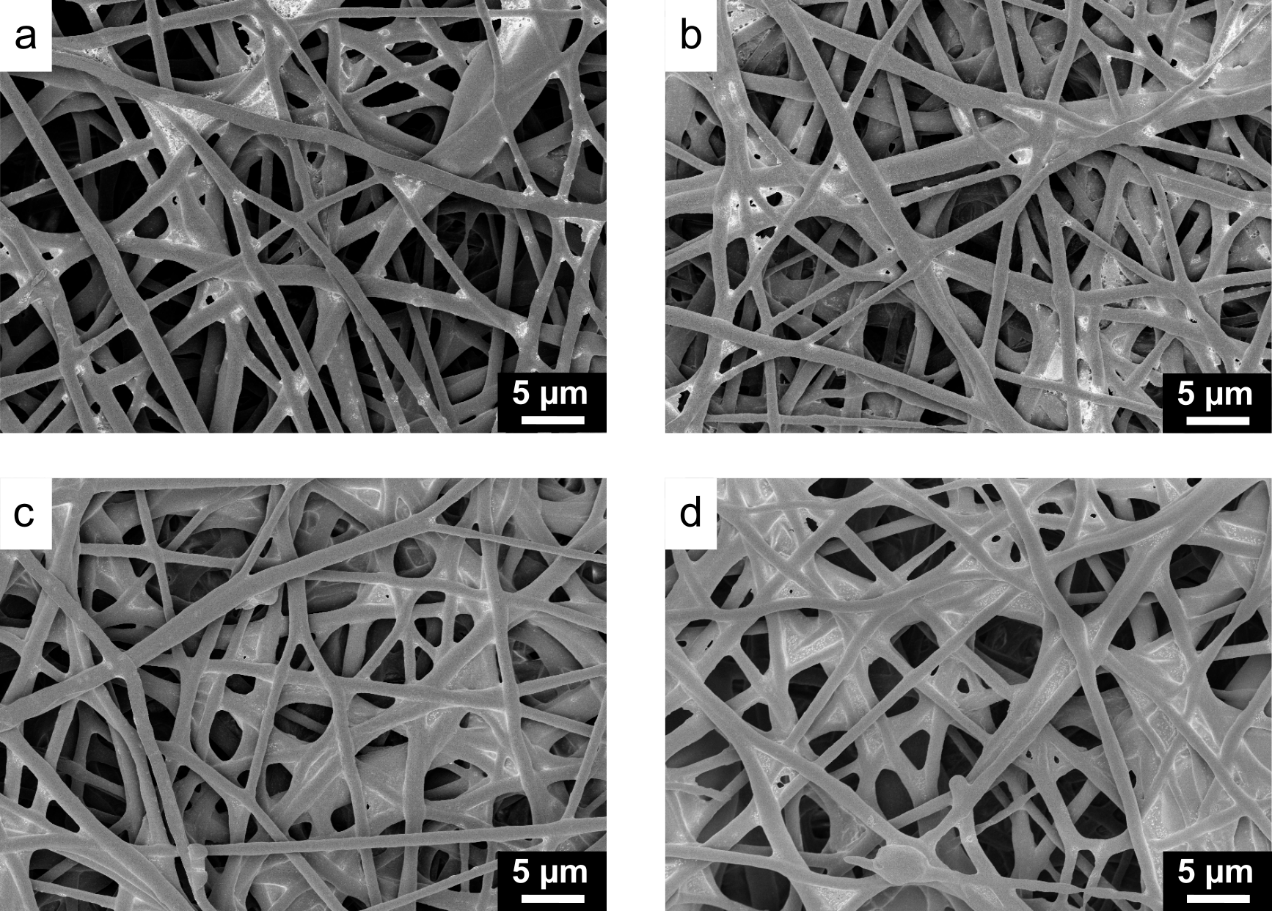


**Fig. S5.** Structural stability of microfibers after various treatments. SEM images of microfiber discs after 40 min of incubation in PBS at (a) 37 °C and (b) 55 °C, and in culture medium (Luria broth) at (c) 37 °C and (d) 55 °C.


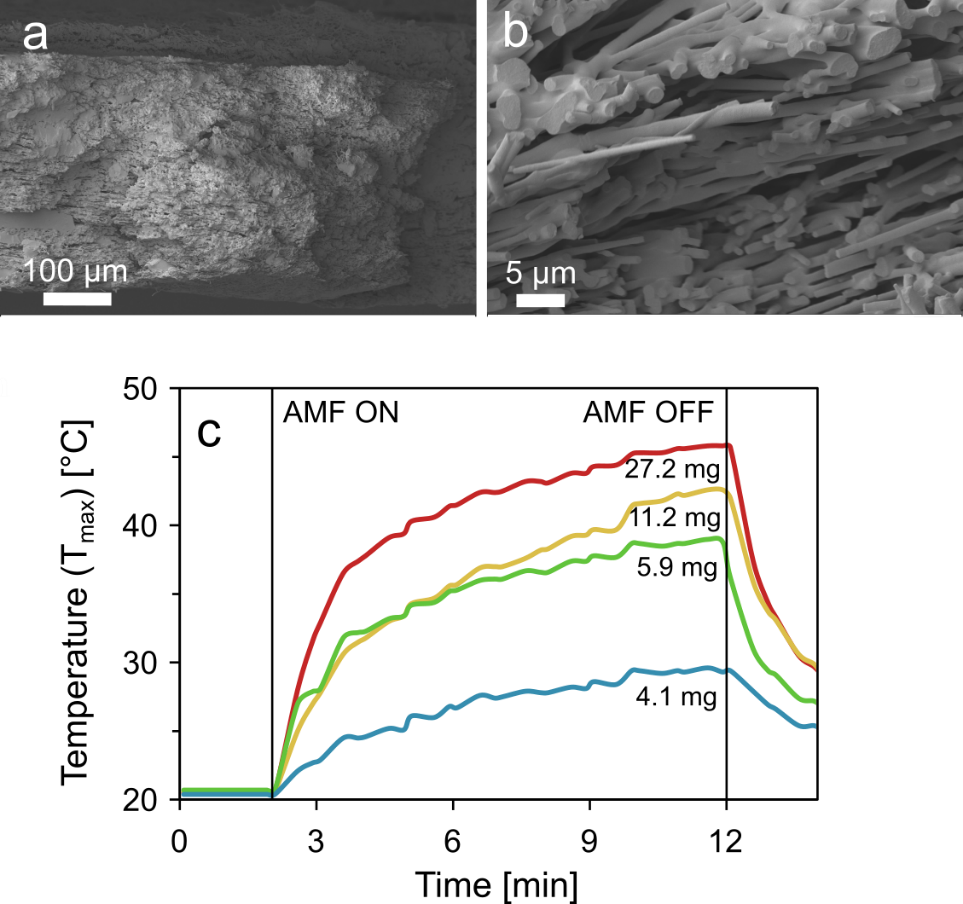


**Fig. S6.** Structural characterization and heating performance of magnetic microfibers. (a,b) SEM images of compacted SPION-loaded PMMA microfibers. (c) Temperature of microfiber discs as a function of AMF exposure time for discs with different weights. The measurements were performed in 100 μL of phosphate buffered saline (PBS) using 8 mm (Ø) microfiber discs. AMF was turned on at t = 2 min and turned off at t = 12 min.


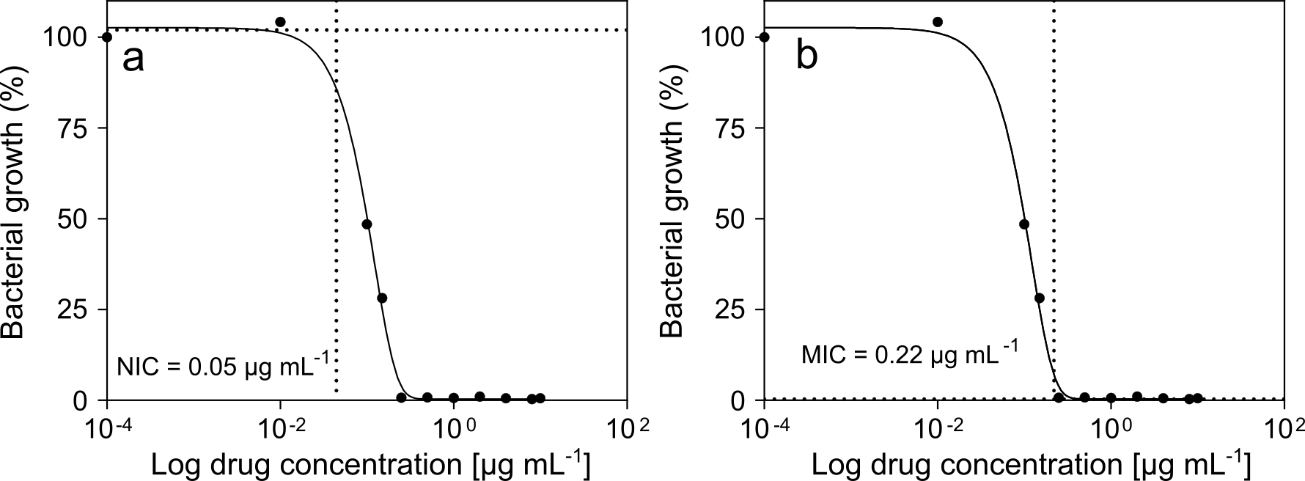


**Fig. S7.** Inhibition of MRSA growth by doxycycline. (a) Non-inhibitory concentration (NIC) and (b) minimum inhibitory concentration (MIC) of doxycycline.


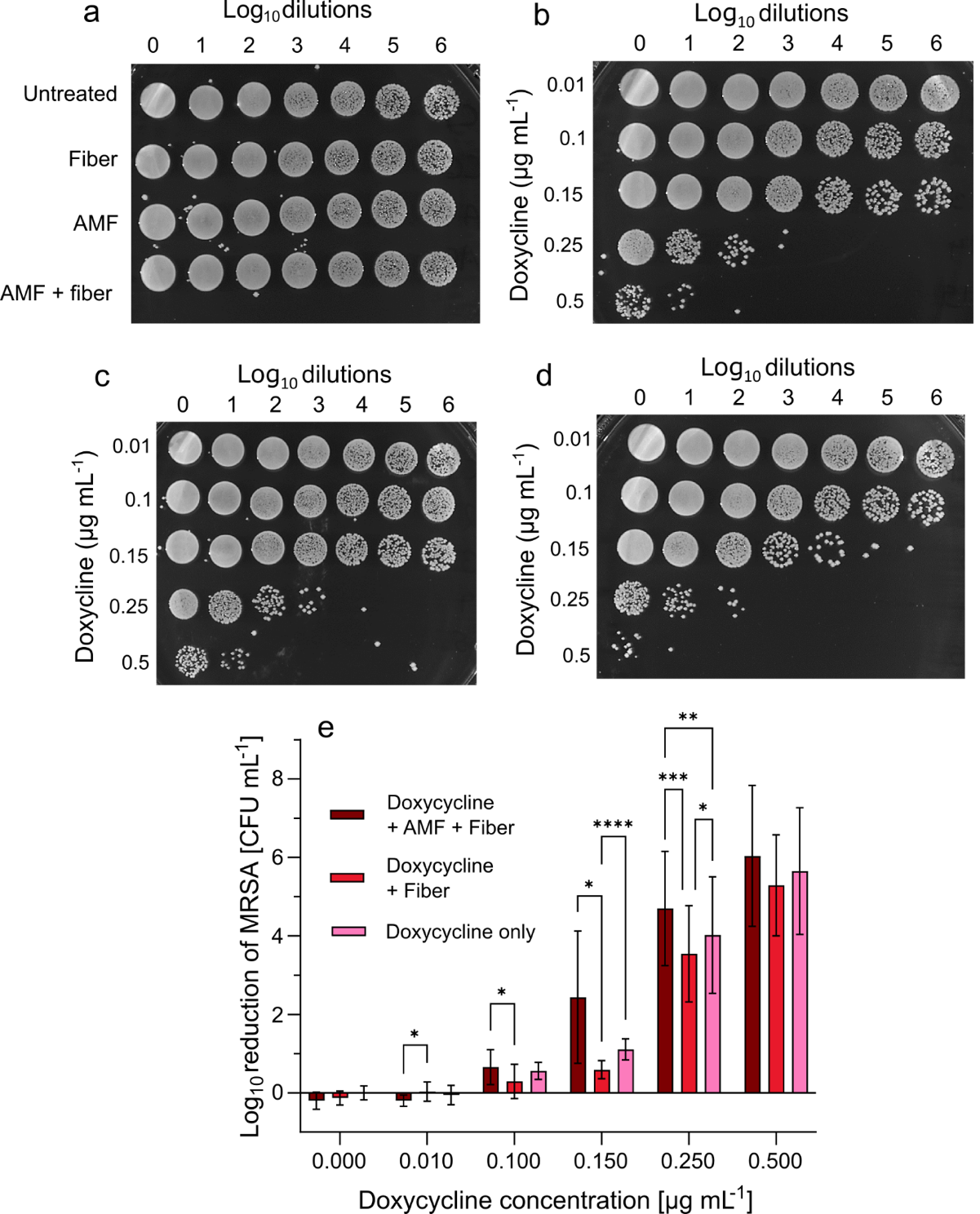


**Fig. S8.** Antibacterial efficacy of magnetic hyperthermia and doxycycline. (a-d) Representative images showing the spot dilution assay of MRSA cultures under various treatment conditions: (a) without doxycycline, (b) doxycycline alone, (c) doxycycline and SPION loaded microfibers, and (d) doxycycline, SPION-loaded microfibers, and AMF (14 mT, 592 kHz, 40 min). Cultures were spotted after 18 h of incubation of samples following treatment. (e) Reduction in MRSA colony forming units (CFU) after treatments. Data represented as mean ± SD (n = 3). A two-way analysis of variance (ANOVA) using Tukey’s multiple comparison test was used to compare different groups. *p* values were calculated as > 0.05 (not significant), ≤ 0.05 (*), ≤ 0.01 (**), ≤ 0.001 (***), and ≤ 0.0001 (****). Pairwise comparisons with *p* values ≤ 0.05 are shown.


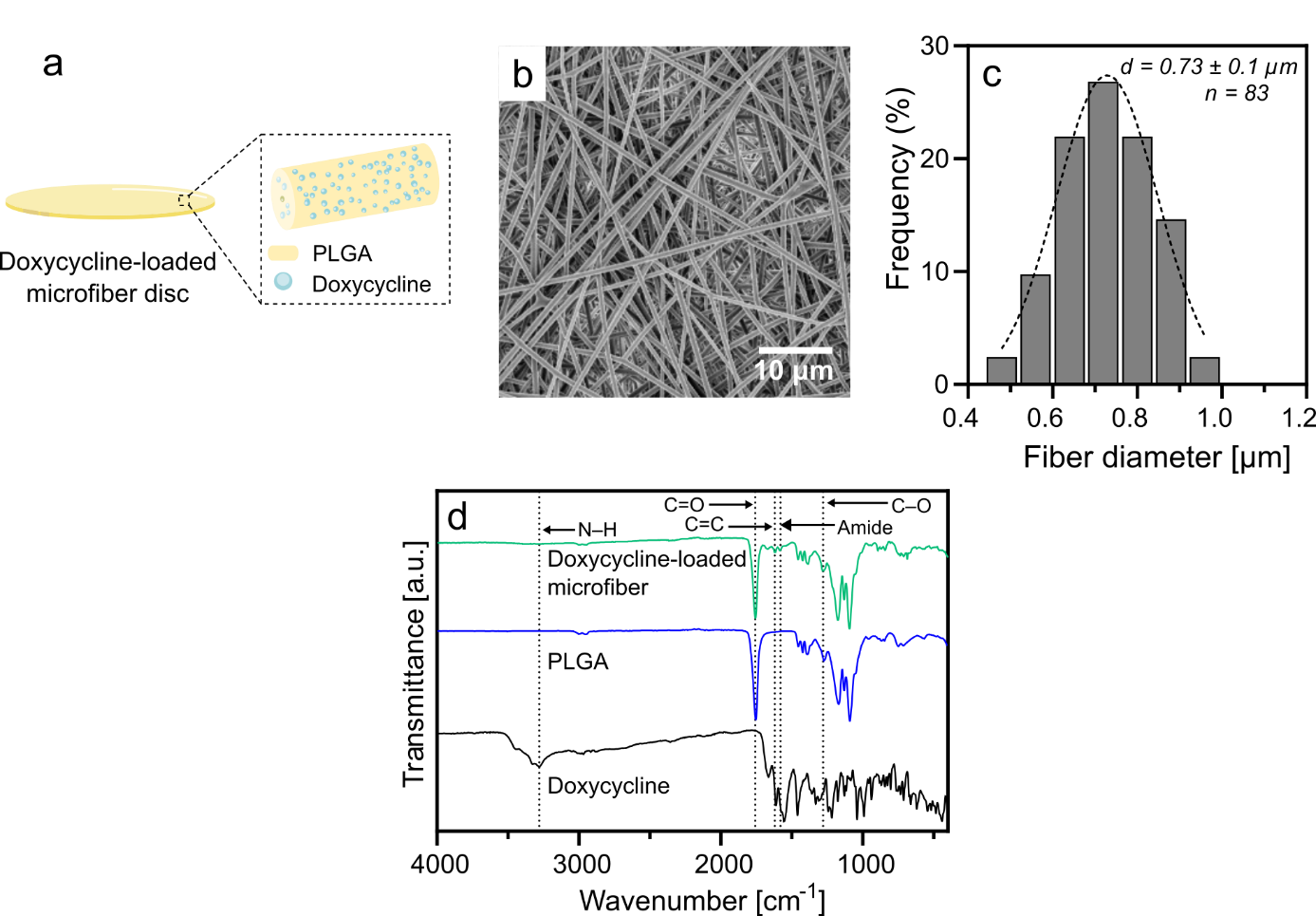


**Fig. S9.** Fabrication and characterization of drug-loaded microfibers. (a) Illustration of doxycycline-loaded poly(lactic-co-glycolic acid) (PLGA) microfibers. (b) SEM image of the compacted doxycycline-loaded microfiber disc and (c) fiber diameter distribution. (d) FTIR spectra of doxycycline-loaded microfibers, PLGA microfibers, and doxycycline.

**Supplementary note.** *Fabrication and characterization of drug-loaded microfibers*

PLGA was used to produce drug-loaded microfibers due to its biocompatibility, biodegradability, and documented use in controlled delivery of doxycycline [1]. The SEM image of the compacted microfiber disc revealed smooth and uniform fibers with minimal defects, and a mean diameter of 0.73 ± 0.11 μm (Fig. S9b and S9c). FTIR spectra confirmed drug incorporation, showing characteristic doxycycline peaks at 1620 and 3280 cm^‑1^ (C=C and N–H), and an amide bond vibration at 1580 cm^-1^, slightly shifted relative to the pure drug, indicating polymer–drug interaction (Fig. S9d) [2].

**Table S1.** Solution composition, viscosity, and process parameters used for electrospinning of microfibers. The concentrations of tributyl citrate (TBC) and SPIONs are with respect to the microfiber weight.

| Solution composition | Viscosity at a sheer rate of 30 s^-1^ [Pa.s] | Feed rate [mL h^-1^] | Voltage [kV] | Needle gauge No. |
| --- | --- | --- | --- | --- |
| PMMA 15 wt% | 0.02 | 2 | 11.0 | 22 |
| PMMA 20 wt% | 0.2 | 4 | 12.1 | 16 |
| PMMA 25 wt% | 0.9 | 4 | 10.5 | 16 |
| PMMA 27.5 wt% | 3.9 | 2 | 9.1 | 16 |
| PMMA 30 wt% | 11.0 | 2 | 8.6 | 16 |
| PMMA 25.4 wt% + TBC  (15 wt%) + SPIONs (23 wt%) | 1.5 | 3 | 11.2 | 22 |

**Table S2.** Summary of mechanical properties of microfibers. The concentrations of tributyl citrate (TBC) and SPIONs are with respect to the microfiber weight.

| Fiber composition | Young’s modulus [MPa] | Ultimate tensile strength [MPa] | Strain at fracture [%] |
| --- | --- | --- | --- |
| PMMA (27.5 wt%) | 29.5 ± 14.8 | 0.36 ± 0.23 | Not determined |
| PMMA (27.5 wt%) + TBC (15 wt%) | 128.5 ± 8.1 | 3.29 ± 0.34 | 7.2 ± 2.3 |
| PMMA (27.5 wt%) + TBC (20 wt%) | 96.8 ± 2.4 | 2.04 ± 0.06 | 7.7 ± 0.8 |
| PMMA (25.4 wt %) + TBC (15 wt%) + SPIONs (23 wt%) | 116.2 ± 33.1 | 1.27 ± 0.38 | 1.2 ± 0.03 |

**References**

[1] C.J. Weng, D. Lee, J. Ho, S.J. Liu, Doxycycline-Embedded Nanofibrous Membranes Help Promote Healing of Tendon Rupture, Int. J. Nanomedicine. 15 (2020) 125. https://doi.org/10.2147/IJN.S217697.

[2] N.-I. Farkas, L. Marincaș, R. Barabás, L. Bizo, A. Ilea, G.L. Turdean, M. Toșa, O. Cadar, L. Barbu-Tudoran, Preparation and Characterization of Doxycycline-Loaded Electrospun PLA/HAP Nanofibers as a Drug Delivery System, Materials (Basel). 15 (2022) 2105. https://doi.org/10.3390/ma15062105.
